# Differential Metabolite Profiles during Fruit Development in High-Yielding Oil Palm Mesocarp

**DOI:** 10.1371/journal.pone.0061344

**Published:** 2013-04-11

**Authors:** Huey Fang Teh, Bee Keat Neoh, May Ping Li Hong, Jaime Yoke Sum Low, Theresa Lee Mei Ng, Nalisha Ithnin, Yin Mee Thang, Mohaimi Mohamed, Fook Tim Chew, Hirzun Mohd. Yusof, Harikrishna Kulaveerasingam, David R. Appleton

**Affiliations:** 1 Sime Darby Technology Centre, Lebuh Silikon, Universiti Putra Malaysia, Serdang, Selangor, Malaysia; 2 Sime Darby Research, Banting, Selangor, Malaysia; 3 Department of Biological Sciences, Faculty of Science, National University of Singapore, Singapore, Singapore; East Carolina University, United States of America

## Abstract

To better understand lipid biosynthesis in oil palm mesocarp, in particular the differences in gene regulation leading to and including *de novo* fatty acid biosynthesis, a multi-platform metabolomics technology was used to profile mesocarp metabolites during six critical stages of fruit development in comparatively high- and low-yielding oil palm populations. Significantly higher amino acid levels preceding lipid biosynthesis and nucleosides during lipid biosynthesis were observed in a higher yielding commercial palm population. Levels of metabolites involved in glycolysis revealed interesting divergence of flux towards glycerol-3-phosphate, while carbon utilization differences in the TCA cycle were proven by an increase in malic acid/citric acid ratio. Apart from insights into the regulation of enhanced lipid production in oil palm, these results provide potentially useful metabolite yield markers and genes of interest for use in breeding programmes.

## Introduction

The tropical perennial tree crop, oil palm (*Elaeis guineensis* Jacq.) has become one of the most productive vegetable oil crops in the world [1] with oil yields averaging approximately 4 tons per hectare per year in Malaysia [2], [3]. However, with growing demand for food and decreasingly available arable land, yield remains an important focus for plantations. In addition, oil yields in excess of 10 ton/hectare/year in trial plots are evidence for further potential increases. Therefore, yield is still the primary trait targeted in oil palm breeding programmes, which primarily use traditional breeding techniques based on iterations of progeny testing and parental selection. With the exception of tissue culture methods [4], [5], the application of other recently developed molecular tools such as DNA-based molecular markers [6]–[8] and most recently, “omics” approaches (genomics, transcriptomics, proteomics and metabolomics) [9]–[12] for oil palm breeding is still in its infancy. However, modern molecular techniques may contribute significantly to traditional breeding methods by providing markers of yield for improved parental and progeny selection and by reducing reliance on extensive field testing and yield recording [13]–[15].

The lipid-rich mesocarp is the main source of oil in the oil palm, producing approximately equal amounts of saturated and unsaturated fatty acids. Overall oil yield is a complex trait controlled by many genes with additive effects. “Omics” technologies that probe the interactions and perturbations in the whole cell system should assist in understanding the causes of yield differences in genetically related commercial populations. While two recent studies [16], [17] have reported valuable insights into the key development stages, transcriptional regulation and carbon partitioning during fruit development in oil palm, investigation into the biosynthetic processes leading to higher oil yield in commercial oil palm populations has not been reported to date.

Analogous to genomics, which defines all genes in a genome irrespective of their functionality, metabolomics seeks to profile all metabolites in a biological sample irrespective of the chemical and physical properties of these molecules [18]. Although targeted phytochemical analysis has long been a fundamental component of plant metabolism research, modern metabolomic profiling can yield more complete and biologically meaningful metabolic information. Metabolite levels can be viewed as the end phenotype associated with valued commodities such as oil, carbohydrates or essential nutrients, and therefore can provide insights into their related biosynthetic processes. Intensive research has been carried out on fruit development in strawberry and tomato using metabolomics [19], [20], but few studies have been reported on oil-bearing fruit such as avocado, olive [21] and in particular, the oil palm [17], [22]. The oil palm fruit is a sessile drupe, and is produced in bunches containing 1000–3000 fruitlets. Oil deposition in the mesocarp starts at about 15 weeks after pollination (WAP) and continues until fruit maturity (20–22 WAP).

Using a metabolomic approach, this study compared the mesocarp metabolite concentrations during critical oil production stages of fruit development between two groups of genetically related oil palm populations that exhibited a 2-fold difference in oil yield in order to identify metabolite markers of increased yield and to provide clues as to what contributes to oil yield at a biosynthetic level. Plants produce various metabolites, ranging from simple primary metabolites to highly complex secondary products [23]. Focused analysis of primary metabolites should reveal important changes in key biosynthetic processes that either lead to or are a result of increased lipid biosynthesis, thereby directing further work on genetic markers for breeding programmes and gene expression studies. No single analytical method can be used to profile accurately all plant metabolites. Hence, this study took a multiple platform approach using gas chromatography-mass spectrometry (GC-MS), liquid chromatography-mass spectrometry (LC-MS) and capillary electrophoresis-mass spectrometry (CE-MS) and employing both untargeted techniques and specific targeting of relevant primary metabolites, including amino acids, carbohydrates, fatty acids and lipids, in palm oil mesocarp.

## Materials and Methods

### Plant material

Each of two screening populations of oil palm plants, one high-yielding and the other low-yielding, consisted of eight individual palm plants. The screening populations were chosen from commercial crosses of Serdang Avenue dura and AVROS pisifera to yield hybrid tenera progeny. The high-yielding group (HY) was identified by their relatively high yields of palm oil after 7 years of yield recording, specifically 10 to 12 tons of palm oil per hectare equivalent per year for each individual. The low-yielding group (LY) had yielded relatively lower amounts of palm oil, specifically 4 to 7 tons of palm oil per hectare equivalent per year. Both groups came from a commercial estate in Carey Island, Selangor, Malaysia and were planted in the same field and of the same age. Fruit bunches were harvested at different developmental stages preceding, during and after the major oil biosynthesis period [2], [16] at 12, 14, 16, 18, 20 and 22 WAP (Figure S1). All fruitlets were separated from the bunches, then 20 were randomly selected from each without bias to location in the bunch. Mesocarp tissues were sliced and snap frozen in liquid N_2_ in order to quench metabolism of the plant tissues and enzyme activity, then stored at −80°C until used for metabolite analysis. Prior to extraction, mesocarp samples were ground to a fine powder using a pestle and mortar.

### Metabolite extraction for GC-MS and LC-MS analysis

The extraction of polar and lipid metabolites from oil palm tissues was carried out in a single integrated procedure. Three technical replicates from each of the 8 HY and 8 LY independent biological replicates were subjected to extraction and analysis. Ground mesocarp samples (100 mg) were extracted with isopropanol (2 mL) containing 0.05% butylated hydrotoluene and heated to 75 °C for 15 min. After allowing the sample to cool, a mixture of chloroform:methanol:water (1∶1∶1, 3 mL) was added along with ribitol (60 µL, 0.2 mg/mL in H_2_O) and phenanthrene (60 µL, 0.2 mg/mL in CHCl_3_) as internal standards. The samples were mixed by vortex for 1 min and shaken at 60°C in a thermomixer at 750 rpm for 1 h, followed by centrifuging at 9000x*g* for 2 min. The supernatant was then removed and the residue was washed again by the addition of chloroform and water (1 mL each), followed by shaking at 60°C in a thermomixer at 750 rpm for 30 min and centrifuging. The combined supernatants were then centrifuged at 9000x*g* at 4°C for 5 min to yield two layers as the polar and non-polar fractions. Equal portions (500 µL) each of polar and non-polar layers were separated into 2 mL tubes. The polar fraction was dried under vacuum while the non-polar fraction was further washed with 500 µL of 1 M KCl before being dried under nitrogen gas. The dried samples were then stored at −80°C until further analysis.

### Moisture and Oil Content Analysis

Moisture content in the mesocarp was determined directly by freeze–drying tissue (100 mg) until a constant weight. Oil content at all the development stages was calculated on the basis of lipid extracted using hexane per dry weight of the tissue.

### Derivatization for GC-MS

Many metabolites contain polar functional groups that are thermally labile at the temperatures required for their separation or are not volatile at all. Therefore, derivatization of the compounds in the polar extracts prior to GC analysis was necessary: Samples were taken from storage and dried under vacuum for 30 min prior to derivatization to remove residual H_2_O. Methoxyamine hydrochloride (120 µL, 20 mg/mL in pyridine) was added and samples incubated at 60°C for 4 h. After that, MSTFA (120 µL, 1% trimethylchlorosilane) was added, followed by shaking at 60°C for 1 h. Since lipid compounds in the non-polar extracts required transesterification in methanol, samples were taken out of storage and dried under nitrogen for 30 min before derivatization. Dried extracts were dissolved in chloroform (100 µL) and methanol (300 µL) with 1.25 M HCl, then incubated at 50 °C for 24 h. Samples were then dried under nitrogen for 2 h. Dried samples were re-suspended with pyridine (70 µL) and MSTFA (30 µL, 1% trimethylchlorosilane) and further incubated at 50°C for 1 h. All samples were left to equilibrate to room temperature before injection into GC-MS for analysis.

### GC-MS analysis

Samples were analyzed using an Agilent 6890N gas chromatograph (GC) coupled with an Agilent 5973i Mass Detector and a 6890 series autosampler. Sample (1 µL) was injected into a programmable injector. For chromatography, a DB5-MS column (15 m×0.25 mm×0.25 µm) was used with helium gas at constant rate of 2 mL/min. GC conditions were as follows: injector temperature, 45°C; detector temperature, 360°C; initial oven temperature, 45°C; detector temperature, 370°C; initial holding time, 1 min; ramping rate, 10°C/min; final temperature, 350°C; final holding time, 16 min; carrier gas (He) flow rate, 2 cm^3^/min; column pressure, 14.5 psi; injection volume, 1 µL. For mass spectrometry, the GC-MS interface temperature was 250°C. MS acquisition conditions were electron impact (EI) ionization at 70 eV, solvent delay 3.5 min, source 230°C, mass range 50–800 da at 4 spectra/s. The MS scan parameters included a mass range of *m/z* 50–800 and a scan interval of 0.5 s.

For lipophilic fractions, helium was used as carrier gas (35 cm/s) with a DB-1 J&W capillary column. The chromatographic conditions were as follows: initial temperature, 100°C for 3 min; temperature rate, 5°C/min; final temperature, 340°C for 12 min; injector temperature, 320°C, split ratio, 1/100. Measured mass spectra were deconvoluted by the Automated Mass Spectral Deconvolution and Identification System (AMDIS) before comparison with library data of reference compounds. The mass spectral data were analyzed using AMDIS software and comparison was made using commercial NIST 05 Mass spectral Library and the Golm metabolome database to identify specific metabolites. GC-MS data was used to analyze sugar content and to cross-validate results for amino acids, organic acids and lipid species.

### LC-MS parameters

LC-MS data were acquired using Accela LTQ Orbitrap instrument (Thermo Fisher, Germany). The LC-MS system (controlled by Xcalibur version 2.0, Thermo Fisher Corporation) was run in gradient mode using an Acquity UPLC® HSS T3 (1.8 µm, 2.1×100 mm; Waters) column set at 45°C. Solvent A was H_2_O (0.1% formic acid v/v) and solvent B was acetonitrile (0.1% formic acid v/v); the flow rate was 0.2 mL/min. The gradient was set as follows: 1% B (0–1.8 min), linearly increased to 10% B (3 min), then increased to 40% B at 20 min–23 min, to 90% B at 26–28 min and to 1%B at 29–35 min. The autosampler temperature was set at 10°C with 3.0 µL injection volume. MS analysis was carried out in positive and negative ion electrospray ionization (ESI) modes of detection. The mass scanning range was 100–2000 *m/z*, while capillary temperature was 300°C, and sheath and auxiliary gas flow rates were 35 and 15 arb (arbitrary units), respectively. The sweep gas flow rate was set at 1 arb and I-spray voltage at 4.5 kV. The resolution was set to 30,000. The capillary voltage and tube lens were set at 40 V and 80 V, respectively, for positive ion mode and at −2.00 V and −47.44 V, respectively, for negative ion mode. The MS/MS spectra of metabolites for identity confirmation were obtained using a collision energy ramp at 35 V. The raw data was processed and compared using Sieve version 1.2 (Thermo Fisher, Alpha Analytical, Malaysia) with the frame time and *m/z* width set at 1.5 min and 0.002 Da, respectively.

### LC-MS lipid species analysis

Targeted profiling of lipid compounds was carried out at Kansas Lipidomics Research Centre, Kansas State University, USA, as per previously published method [24].

### Targeted profiling of polar metabolites using CE-MS

Targeted profiling of 108 primary metabolites using CE-MS was carried out by Human Metabolome Technologies, Inc (HMT), Japan, as per previously published method [25].

### Statistical analysis

Principle component analysis (PCA) and Orthogonal Partial Least Square-Discrimination Analysis (OPLS-DA) using Simca-P version 12 (Umetrics) were used to identify metabolites that reflect the differences between HY and LY palm tree groups. The t-test algorithm of Excel 2000 (Microsoft) was used for determining significant difference (P<0.05).

## Results and Discussion

The differences in oil yield among the two segregated populations of the selected oil palm trees, planted in the same location, indicated that oil yield differences would most likely be due to genetic variation. Oil yield and fruit bunch analysis data collected for individuals revealed an almost 2-fold overall yield differential between the low-yielding (LY) and high-yielding (HY) groups with average yields of 40.5 and 78.1 kg/palm/year, respectively. Closer inspection of the fruiting characteristics leading to the dramatic increase in oil production for the HY group indicated simultaneous increases in the number of bunches produced per year, the average mesocarp mass per bunch and the oil content of the mesocarp (Table 1). This suggests a number of factors working in concert to produce significantly higher overall oil yield, while the lack of negative correlation between the number of bunches produced and size of bunches suggests that photosynthetic precursors to oil production may not be limiting in this case [26].

**Table 1:** Harvested ripe bunch oil yield data and fruit bunch characteristics for LY and HY oil palm populations studied. Data collected throughout seven years of recording.

	LY (n = 8)			HY (n = 8)		
	Mean	Min	Max	Mean	Min	Max
Total oil yield (kg/palm/year)	40.5	30.8	49.0	78.1	72.7	86.4
No. of bunches (/year)	21`	17	27	25	21	28
Bunch weight (kg)	9.2	7.2	11.7	11.0	10.2	12.1
Mesocarp weight (kg/bunch)	4.3	3.3	5.6	5.8	5.2	6.6
Oil composition (% wet wt. mesocarp)	46	37	55	55	52	61

**Figure 1:**
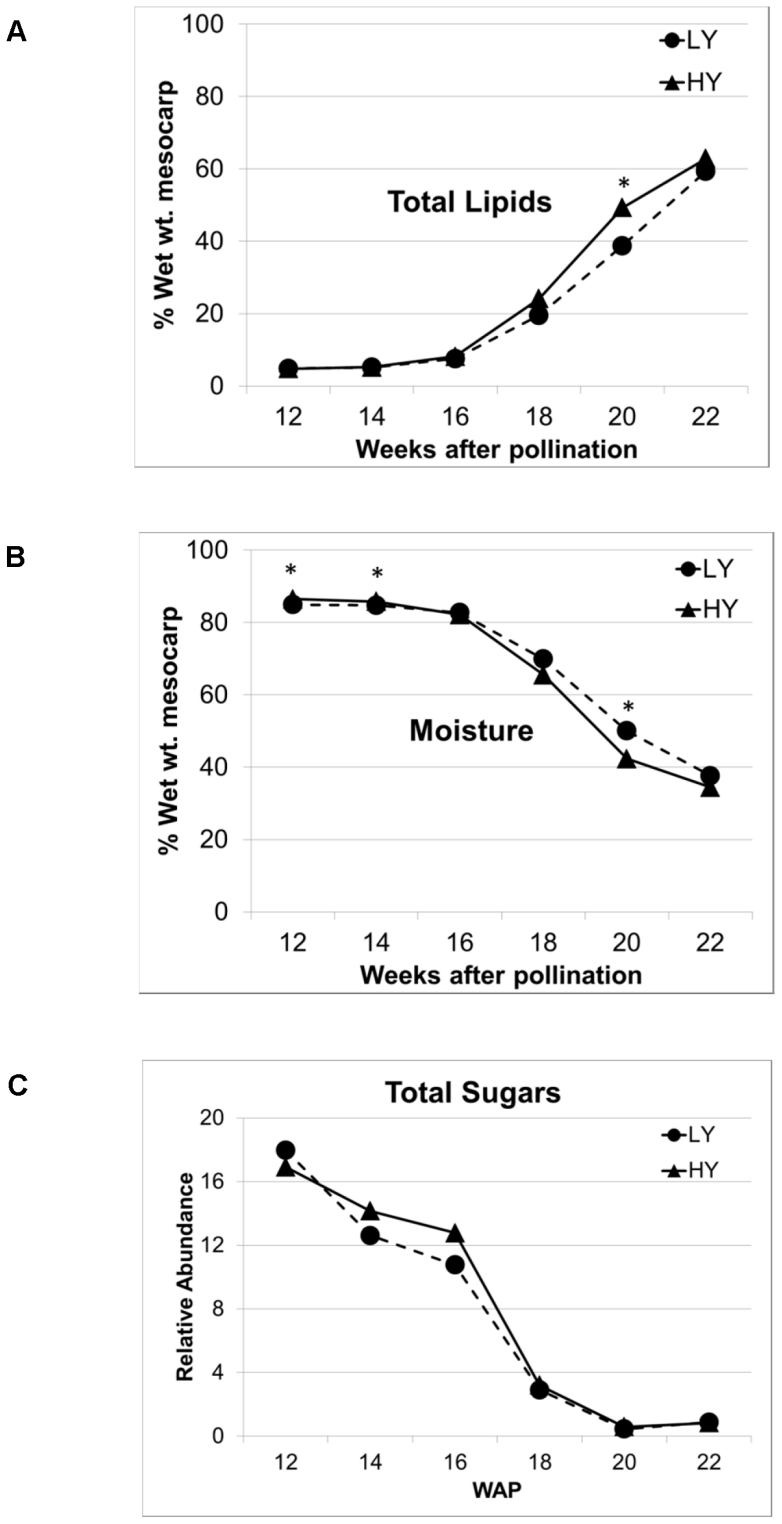
Total lipid, moisture and sugar contents of mesocarp tissue during development. High-yielding (HY) and low-yielding (LY) palm fruit mesocarp tissue composition during the critical period of oil palm fruit development (n = 8): a) total lipid, b) moisture content, and c) content of major sugar species. Error bars show ± 1σ; *, *p*-value for difference <0.05.

Fruitlets from bunches sampled during the critical stages of oil production were initially analyzed to determine lipid and moisture content before, during and after the major lipid production stages [16]. Figure 1 shows the significant increase in lipid content (Figure 1a) between 16 and 18 WAP, essentially replacing the moisture content of the mesocarp (Figure 1b) and reaching 49% and 39% of the mesocarp wet weight for HY and LY groups, respectively, at optimal fruit harvest time of 20–22 WAP. Conversely, with lipid production, the sugar content (Figure 1c) along with overall polar extractable components of the fruit decreased to very low levels by 18 WAP with minimal difference between HY and LY groups. These results were consistent with the findings of several previous studies investigating the developmental stages of oil palm fruit that highlighted the three major developmental events of oil production: lag period preceding lipid biosynthesis, 12–16 WAP; fruit maturation/oil biosynthesis, 16–20 WAP; and fruit ripening/maturation, 20–22 WAP [2], [16], [17], [27], [28].

**Figure 2:**
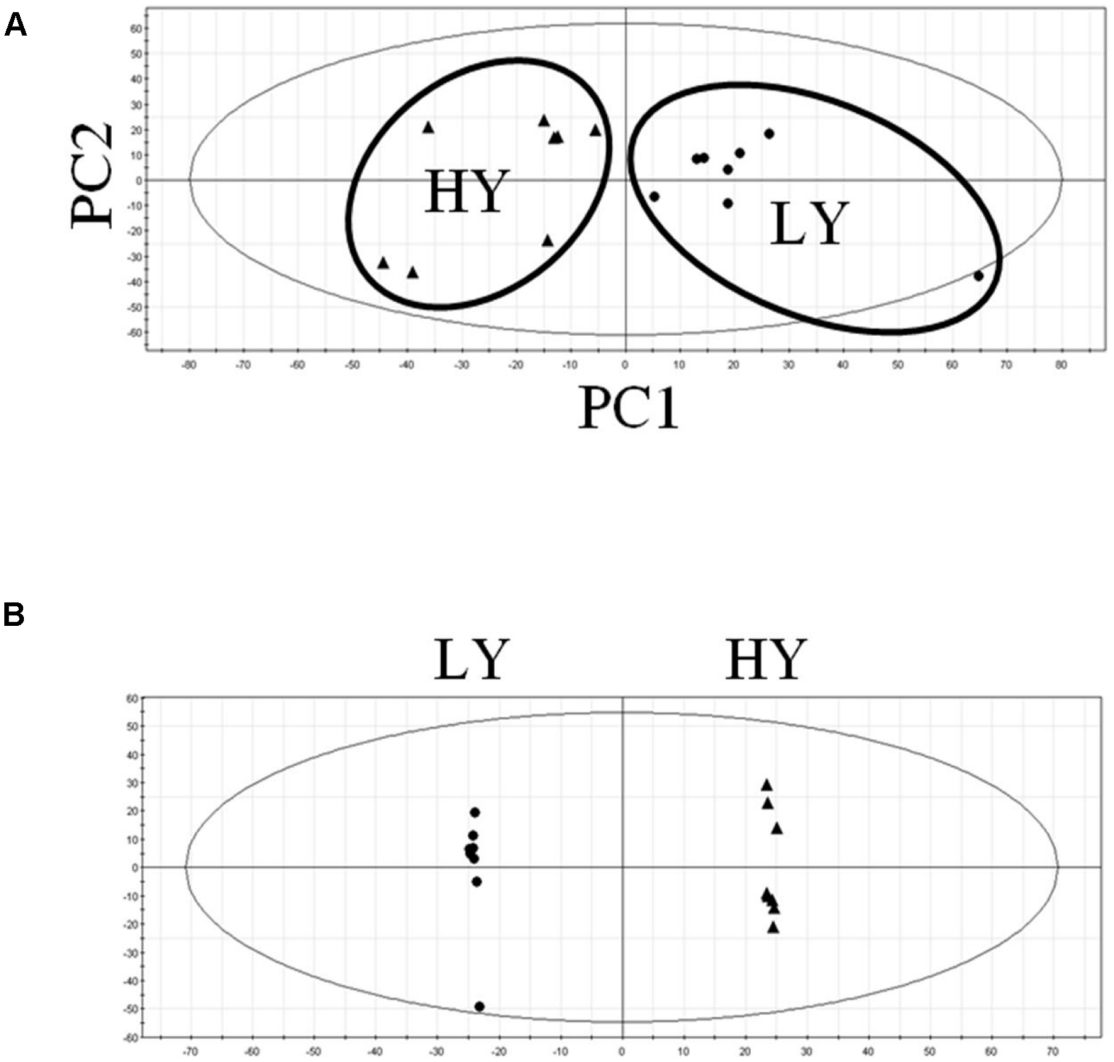
Multivariate analysis of mesocarp metabolites in HY and LY palms at onset of lipid biosynthesis. Plots of a) principal component analysis (PCA) scores and b) orthogonal partial least squares discriminant analysis (OPLS-DA) scores of HY (▴) and LY (•) mesocarp polar extract metabolites at 16 WAP obtained from untargeted analysis using LC-MS.

For each developmental time point, the polar mesocarp extracts of individual palms in the HY and LY groups were profiled using an untargeted LC-MS approach in order to gather information about as many metabolites as possible [29], [30]. Using multivariate analysis of the data, it was found that the level of distinctiveness of the HY and LY samples varied across the time period studied. Polar extracts of samples at 12, 14 and 16 WAP clustered well into the two groups of HY and LY, indicating different metabolite profiles associated with the two groups. However, as the overall concentration of polar components in the mesocarp decreased at the commencement of lipid production (>16 WAP), the distinctiveness was less evident. The highest differentiation of HY and LY palm polar mesocarp metabolites was found to be immediately preceding the onset of lipid biosynthesis at 16 WAP (Figure 2), indicating significant detectable differences between the levels of polar metabolites in the mesocarp of higher yielding palm trees and those of lower yielding ones, particularly in the early stages of lipid biosynthesis.

**Figure 3:**
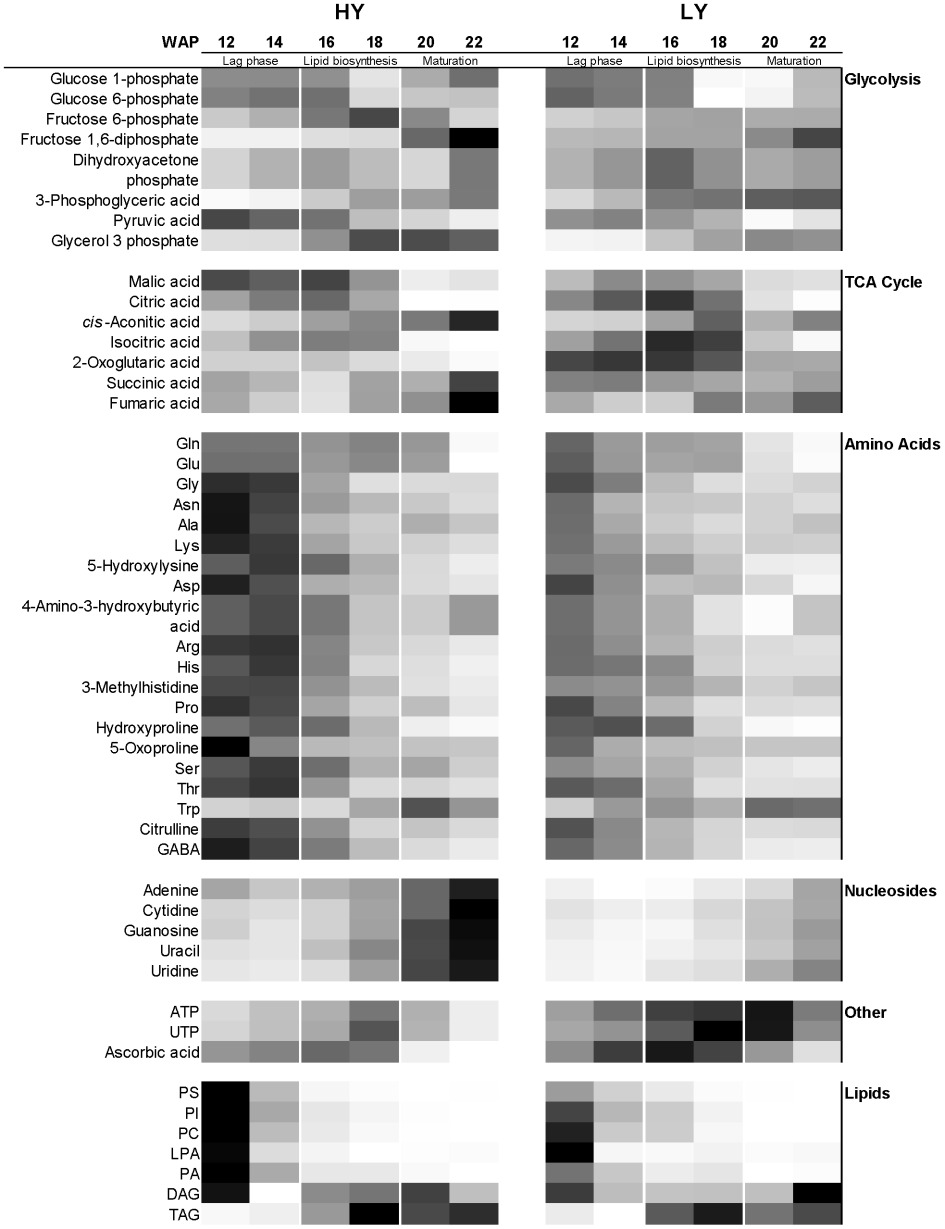
Heatmap of important primary metabolite concentrations during HY and LY palm mesocarp development. Heatmap of averaged and normalized mesocarp metabolite concentrations in key biosynthetic and cellular pathways of HY and LY oil palm groups at 12-22 WAP (n = 8). Scale from white (lowest concentration) to black (highest concentration) within each metabolite class.

Further analysis using orthogonal partial least squares discriminant analysis (OPLS-DA), as shown in Figure 2b at 16 WAP, increased the resolution of the HY and LY metabolite profile differences and allowed the identification of a number of differential metabolites at all developmental stages analyzed. Several metabolites were identified as markers of higher oil yield were elucidated based on their MS/MS fragmentation profiles; they included primary metabolites such as arginine, homo-arginine, vanillactic acid and several organic acids. Subsequently, extensive analysis of 108 metabolites involved in key biosynthetic and cellular processes was conducted using CE-MS. This investigation revealed significant differences across a number of the key metabolite classes at different stages of fruit development in HY and LY palm samples, including lipids, glycolysis, TCA cycle organic acids, amino acids and nucleosides (Figure 3).

### Differential mesocarp lipid species of high yielding oil palm

**Figure 4:**
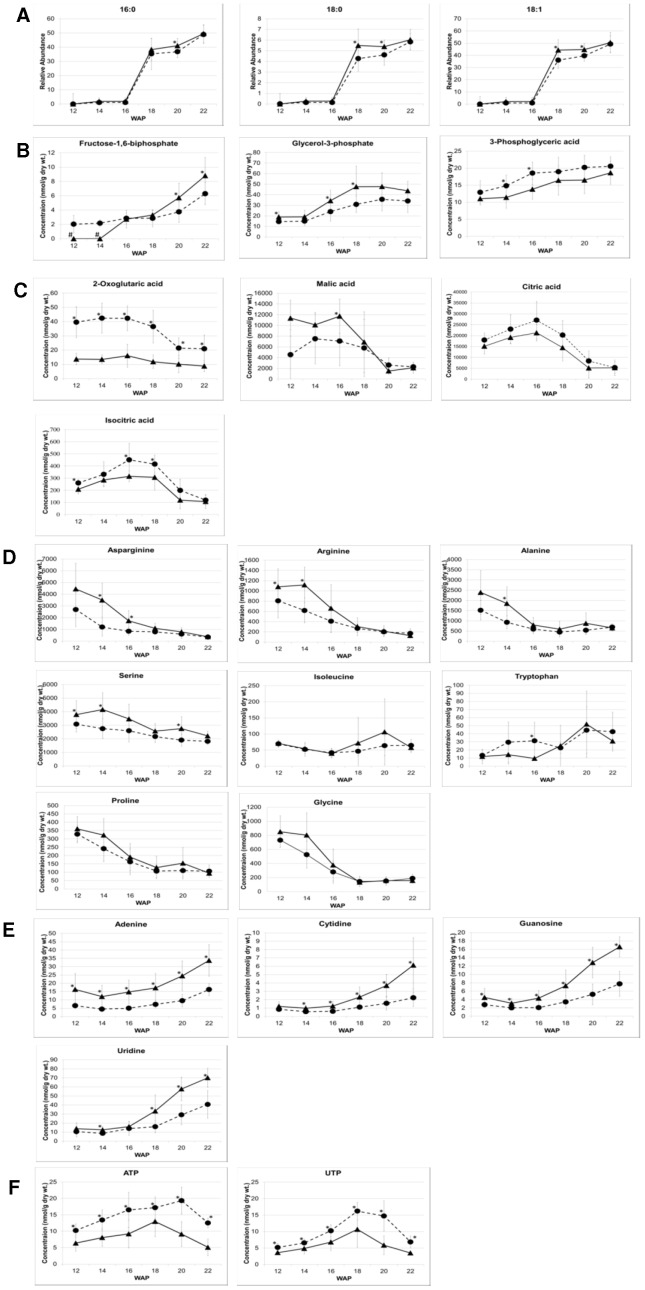
Differential metabolite profiles across key pathways during development of HY and LY palm mesocarp. Significant differential mesocarp concentrations of selected metabolites in HY (▴) and LY (•) palms during 12–22 WAP (n = 8) for a) fatty acids, b) glycolytic pathway, c) TCA cycle, d) amino acids, e) nucleotides, f) other selected metabolites. Error bars show ± 1σ; *, *p*-value for difference <0.05; #, not detected.

The HY and LY groups investigated in this study exhibited a difference of up to 20% in mesocarp oil concentration at the time of harvesting (∼20 WAP) (Table 1). This trend could also be seen using GC-MS analysis of the fatty acid species concentrations during fruit development, as seen in Figure 4a. Significant differences were observed in the concentrations of the three major palm oil fatty acid species, 16∶0, 18∶0 and 18∶1, during lipid biosynthesis (18–20 WAP) (Table S1). The differentials were less clear after this point, probably due to over-ripening processes. Further investigation using LC-MS of the mesocarp lipids, including phospholipid species in HY and LY palm samples (Table S2), revealed that the largest differences were in the intermediate diacylglycerol (DAG) concentrations as well as several phospholipid species (Figure 3). Lipid biosynthesis in the mesocarp occurs by two distinct pathways: the first one is the Kennedy pathway, which relies on a sequential acylation process of fatty acids on a glycerol-3-phosphate backbone provided by glycerol-3-phosphate dehydrogenase (G3PDH) from dihydroxyacetone phosphate (DHAP), while the second pathway relies on acyl exchange between lipids and involves phospholipid diacylgycerol acyltransferase (PDAT). DAG is the final intermediate before the formation of triacylglycerol (TAG) in the endoplasmic reticulum (ER). Significant differences in EST levels between oil palm and its non-oil producing relative, date palm, that have been reported for certain isoforms of phosphotidic acid (PA) phosphatases and phosphotidyl choline (PC)-related enzymes [17] may also be involved in the differences observed here between HY and LY oil palm. However, the most significant differences in metabolite concentration were observed in the polar metaboite species involved early in the lipid biosynthesis pathway and other primary metabolites, as described below. This observation somewhat parallels that which has also been reported in the comparison between oil and date palms that indicated similar transcription levels of enzymes involved in TAG assembly between the oil palm and sugar-producing date palm, with the majority of differences being earlier, involving the supply of pyruvate and fatty acid biosynthesis.

### Differential mesocarp metabolites in glycolysis of high-yielding oil palm

The intermediate metabolites involved in glycolysis exhibited varied trends between HY and LY groups during fruit developmental stages. The early intermediates (glucose, glucose-1-phophate and glucose-6-phosphate) exhibited similar concentrations and trends between HY and LY, while fructose-6-phosphate appeared at higher concentrations in HY during lipid biosynthesis (Figure 3). Levels of fructose-1,6-biphosphate appeared to be initially lower in HY, preceding the start of lipid biosynthesis but increased later during maturation and exceeded the levels measured in LY samples at 20 WAP (Figure 4b). The most significant differences were in concentrations of glycerol-3-phophate and 3-phosphoglyceric acid, with glycerol-3-phosphate being higher and 3-phosphoglyceric acid being lower in the HY group compared to the LY group throughout the last stages of fruit development studied here (Figure 4b). The concentration of pyruvic acid was observed to be marginally higher in HY palms before lipid biosynthesis started at 16 WAP. Glycerol-3-phsophate is the building block that is acylated with fatty acids to produce lipid (TAG) molecules. It has been demonstrated in previous studies that diversion of carbon flux through glycolysis to produce more glycerol-3-phosphate can lead to higher levels of lipid production in seed oils [31], [32]. Vigeolas *et al.* showed that a three- to four-fold increase in the levels of glycerol-3-phosphate in *Brassica napus* by increasing the activity of G3PDH resulted in a 40% increase in the seed lipid content [32], [33]. In sunflower seeds, it has shown that the two main metabolic processes related to variations in oil content between different lines were glycolysis and amino acid metabolism [34], [35]. While Troncoso-Ponce *et al*., by comparing the activities of phosphoglycerate kinase and phosphoglycerate enolase in sunflowers with standard and low seed-oil content, showed that both enzymes were found to be related to the oil production levels [36]. The comparison of gene transcription between oil palm and date palm by Bourgis *et al*. revealed significant differences in only two of the enzymes in this pathway: levels of both phosphofructokinase (PFK) and pyruvate kinase (PK) were elevated in oil palm compared to those in date palm and have been suggested to be responsible for the greatly increased flux through to pyruvate for fatty acid biosynthesis [9], [17]. Further investigation of the activity and transcription levels of the glycolysis enzymes in the HY population from this study may explain the differences in the metabolite intermediates observed as well as their contribution to overall increased lipid production.

### Differential mesocarp organic acids involved in the TCA cycle in high-yielding oil palm

**Figure 5:**
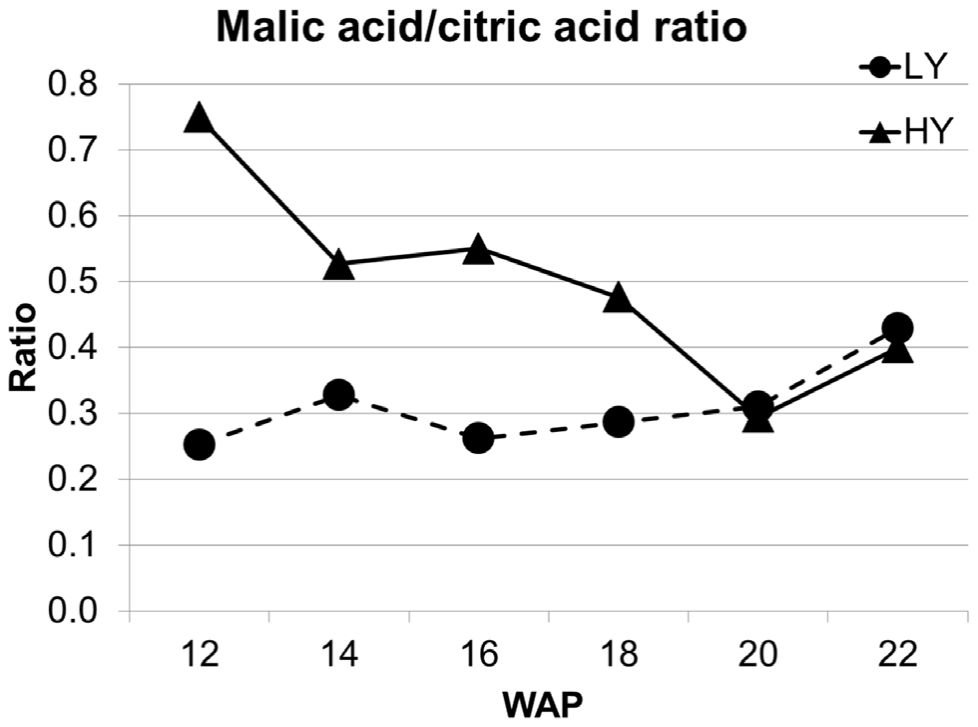
Differences in malic acid/citric acid ratios in HY and LY palms during lipid biosynthesis. Malic acid/citric acid ratios of HY and LY oil palm mesocarp samples during 12–22 WAP (n = 8).

Citric acid, isocitric acid (Figure 3 and Table S3) and, more markedly, 2-oxoglutaric acid all appeared at lower concentrations in the HY group of palms just preceding and during the early stages of lipid biosynthesis (14–18 WAP) while malic acid exhibited a very different concentration profile between the HY and LY groups, maintaining a significantly higher concentration in HY palms from 12 WAP through to the mid-point of lipid biosynthesis (18 WAP) when it dropped to a level similar to that of the LY group (Figure 4c). The differing trends of malic acid and citric acid concentration in HY and LY palms can be clearly seen in Figure 5, where the ratio of malic acid to citric acid is clearly higher in HY palms from 12 WAP until when lipid biosynthesis reaches a maximum at 18 WAP. High malate to citrate ratio during lipid biosynthesis in the mesocarp of olive has been reported previously [37] and appears also to be an important feature in the higher oil-producing ability in the HY oil palm population investigated here. Malic acid has also been shown to be crucial for fatty acid synthesis in developing castor endosperm [38], while the relatively low concentrations of isocritric acid and 2-oxoglucaric acid observed in HY palms during lipid biosynthesis is likely to result from higher utilization of acetyl-CoA for fatty acid production and possibly of other major biosynthetic precursors leading to amino acid/protein production.

### Differential levels of amino acids in mesocarp of high yielding oil palm

Overall it was found that the concentrations of most amino acids declined from 12 to 22 WAP with the highest concentrations observed before commencement of lipid biosynthesis, excluding Trp (Figure 3 and Table S3). Several amino acids were found to have significantly higher concentrations in the HY group from 12–16 WAP, including asparagine, alanine, proline, arginine, serine and glycine (Figure 4d). The most notable deviations from this trend were isoleucine and tryptophan, which showed concentrations in the HY group that were comparable or at lower levels than those in the LY group. Interestingly, the period of fruit development with relatively lower tryptophan concentration in HY palms coincides with the period with the highest concentration of auxin (IAA), as reported by Tranbarger *et al*.[16] In a previous study [35] four enzymes associated with amino acid metabolism were highly up-regulated in low-oil sunflower lines. This study showed that in oil palm, higher oil yield was associated with higher amino acid concentration preceding lipid production (12–14 WAP). The accumulation of amino acids at this stage could possibly support production of the proteins necessary for lipid biosynthesis and cell division that are required later during maturation and mesocarp development. Fruit bunch analysis of the palms used in this study showed a 35% larger mesocarp mass per bunch on average for the HY group compared to the LY group (Table 1), suggesting that mesocarp development is a major driver of increased oil yield. Interestingly, the comparison of oil palm with date palm has also revealed significantly higher concentrations of amino acids in oil palm compared to its non-oil-producing relative [17]. The higher levels of amino acids associated with high oil yield observed in this study indicates that regulation of amino acids plays a crucial role in optimum lipid biosynthesis and fruit maturation.

### Differential levels of nucleosides in mesocarp of high-yielding oil palm

Nucleosides such as adenine, cytidine, guanosine, uracil and uridine were all found to be significantly higher in HY during the later stages of fruit development (18–22 WAP) as seen in Figure 3 and Figure 4e. Overall, the concentration of these nucleosides increased during fruit development to a maximum at 22 WAP (Figure 3). With the exception of adenine (Figure 4e), less significant differences were observed between nucleoside concentrations of HY and LY palms at 12–18 WAP–a trend that is apparently contrary to amino acid concentrations. Purines and pyrimidines are important building blocks for nucleic acids in addition to being indirectly involved in a number of other biochemical processes, including sucrose and cell wall polysaccharide metabolism [39] and lipid production [40], [41]. The possible involvement of purines and pyrimidines in increased oil yield seen here warrants further investigation.

### Other differential mesocarp metabolites in high-yielding oil palm

ATP and UTP were found to be lower in HY palms (Figure 4f) throughout the time points studied, but most markedly during the period of highest lipid biosynthesis (16–20 WAP). Their low concentrations are most likely a result of increased energy demand required to produce the observed differences in lipid production levels in the HY palms, a result similar to that found in a study investigating differences in carbon partitioning between starch and oil in oat cultivars (*Avena sativa*) [41]. Ascorbic acid was also found to be lower in HY palms than in LY palms (Figure 3). While ascorbic acid is known to be an important antioxidant involved in other metabolic functions in plants, such as photosynthetic electron transport, synthesis of plant growth substances, cell wall synthesis and expansion, modulation of hormone signaling and promotion of cell division [42], the significance of the differential levels of ascorbic acid in HY and LY palms is still unclear.

## Conclusion

The study of metabolite profiles during fruit development in two genetically related populations of oil palms in a single location displaying a 2-fold difference in oil production revealed global changes in, or regulation of, amino acid and nucleoside levels in the stages of fruit development spanning lipid biosynthesis. It is possible that the higher levels of amino acids (12–16 WAP) and nucleosides (18–22 WAP) are associated with protein biosynthesis preceding and during oil biosynthesis and later fruit expansion to support oil production, aligning well with current knowledge of the fruit development stages [16]. Differences in the metabolites that are more directly linked to lipid production in the glycolysis pathway and TCA cycle exhibited more complex differentials. This study found interesting divergence in carbon flux away from pyruvate and towards glycerol-3-phosphate as well as significant increases in the malate to citrate ratio preceding and during the lipid biosynthesis periods of fruit development. Concordant with the apparent divergence of flux towards glycerol-3-phosphate, lower triosephoshate isomerase protein levels were detected in the mesocarp samples of HY palms compared to LY during lipid biosynthesis [43]. Simultaneous increases in production of glycerol-3-phosphate through glycolysis and diversion of carbon utilization for acetyl-CoA from the TCA cycle could be significant drivers of increased lipid production in oil palm.

## Supporting Information

Figure S1 The different developmental stages of oil palm fruitlets.
(DOCX)

Table S1 GC-MS analysis of fatty acid species concentrations during fruits development.
(XLSX)

Table S2 Polar and non-polar lipid molecular species of oil palm fruits from LC-MS analysis.
(XLSX)

Table S3 Semi-quantitative raw data and normalized data of targeted compound from CE-MS analysis.
(XLSX)
