# Supplementary material for: Differential Metabolite Profiles during Fruit Development in High-Yielding Oil Palm Mesocarp
Source: PLoS One. 2013 Apr 11;8(4):e61344. doi: 10.1371/journal.pone.0061344 (PMC3623811; doi:10.1371/journal.pone.0061344)
Supplement: Figure S1 — The different developmental stages of oil palm fruitlets. (DOCX) [file pone.0061344.s001.docx]

1. (B) (C)

**Supporting Figure S1.** **The different developmental stages of oil palm fruitlets.** Photographs of oil palm fruitlets collected at (A) 14, (B) 18, and (C) 22 weeks after pollination.
